# Supplementary figures and images for: The role of mitophagy in the development of chronic kidney disease
Source: PeerJ. 2024 Apr 25;12:e17260. doi: 10.7717/peerj.17260 (PMC11056108; doi:10.7717/peerj.17260)

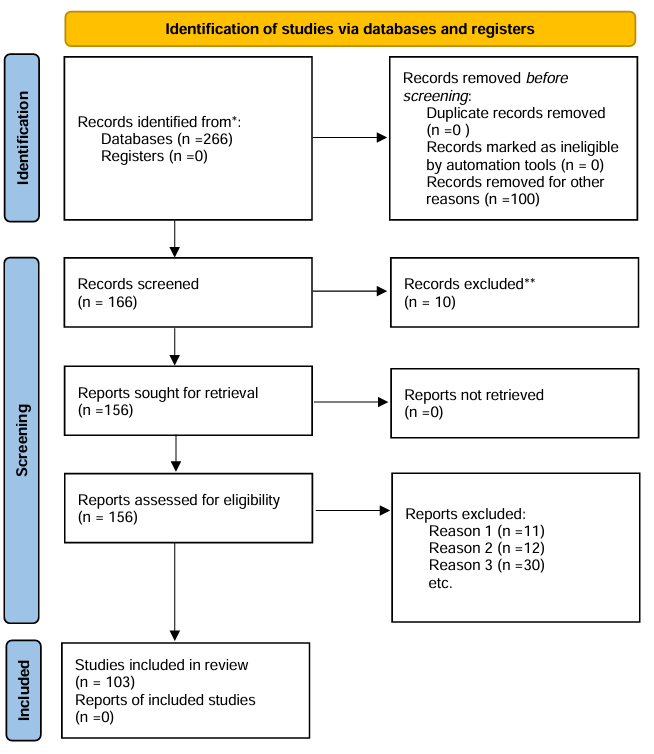

Supplement: Supplemental Information 1 [file peerj-12-17260-s001.png]
